# Supplementary material for: Compound Capecitabine Colon-Targeted Microparticle Prepared by Coaxial Electrospray for Treatment of Colon Tumors
Source: Molecules. 2022 Sep 3;27(17):5690. doi: 10.3390/molecules27175690 (PMC9457672; doi:10.3390/molecules27175690)
Supplement: Supplementary file 1 [file molecules-27-05690-s001.zip › molecules-1842801-supplementary.pdf]

Compound Capecitabine Colon-Target Microparticle Prepared by Coaxial Electrospray for Treatment of Colon Tumors

Table S1. EE_{CAP}, EE_{OSI}, DL_{CAP}, DL_{OSI}, and particle size at different drug concentrations.

Drug Concentration	EE_{CAP} (%)	DL_{CAP} (%)	EE_{OSI} (%)	DL_{OSI} (%)	Particle Size (μm)
0.1	95.6±1.21	1.05±0.13	95.3±1.17	1.03±0.17	1.87±0.131
0.3	94.4±1.67	3.08±0.21	94.8±1.54	3.11±0.22	1.95±0.164
0.5	92.9±1.52	4.93±0.28	93.1±1.49	4.95±0.27	2.09±0.172
0.7	83.1±4.61	5.92±0.41	83.5±3.76	5.96±0.43	2.47±0.237
0.9	79.7±5.73	7.17±0.56	80.2±5.38	7.19±0.51	2.75±0.281

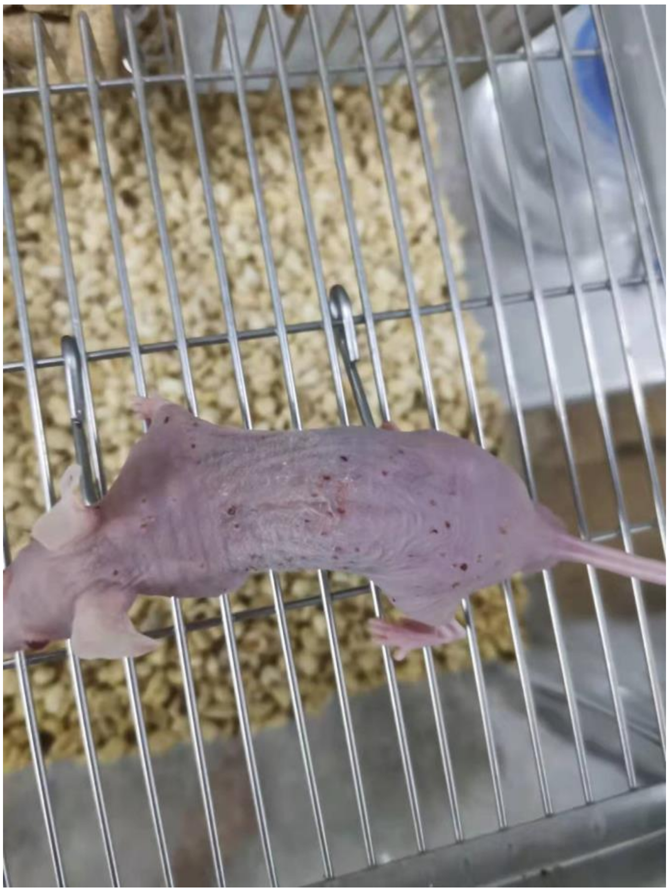

Figure S1. Red spots on the skin of nude mice in free drug groups.

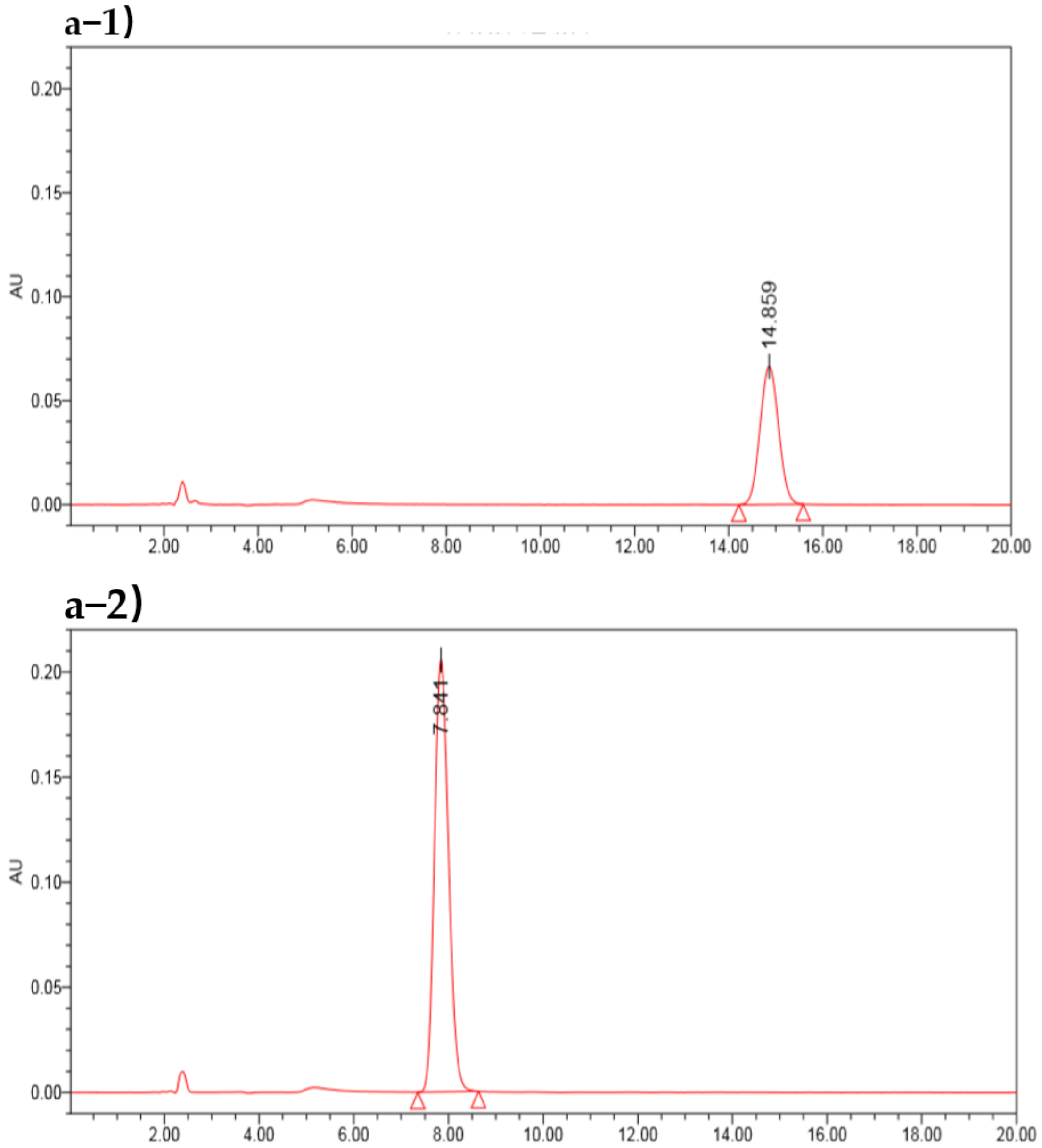

Figure S2. The HPLC of CAP and OSI (a−1. CAP; a−2. OSI).
